# Eruptive Process in Children with Osteogenesis Imperfecta

**DOI:** 10.1007/s00223-025-01345-1

**Published:** 2025-02-07

**Authors:** Clara Sandibel Garcete Delvalle, M. Joaquín De Nova García, María Rosa Mourelle Martínez

**Affiliations:** Faculty of Dentistry, Complutense University of Madrid, Madrid, Spain

**Keywords:** Alveolar eruption, Osteogenesis Imperfecta, Bisphosphonates, Root resorption, Dental exfoliation, Dental development, Permanent dentition, Primary dentition, Panoramic radiograph

## Abstract

Osteogenesis imperfecta (OI) is a hereditary disorder characterized by bone fragility and skeletal abnormalities. The administration of bisphosphonates (BPs) in children with OI increases bone density. This antiresorptive inhibits osteoclast action, thus altering physiological processes, in which osteoclasts play important roles, such as the eruptive process. The aim of this investigation was to study the eruptive process (dental development of permanent dentition, resorption of temporary dentition, and alveolar eruption of the first permanent molar) in children with OI medicated with BPs and to compare the results with those of a control group. In total, 34 panoramic radiographs of children with OI [mean chronological age of 8.43 (± 1.77)] who were medicated with BPs for a period of one year or more were studied and 367 panoramic radiographs of healthy children [mean chronological age of 9.19 (± 1.62)] were used as controls. The Demirjian method was used to study the dental development of the seven permanent teeth in the third quadrant. Alveolar eruption of the first permanent molar was considered when perforation of the alveolar bone was produced. The Haavikko method was used to study the root resorption of the five primary teeth in the third quadrant, and software (PixelStick®) was used to measure the lengths of the mesial and distal roots of the primary molars. The cumulative dose of BPs was obtained by mathematically calculating the total dosage received (mg)/weight (kg) and multiplying the relative potency of the medication. The Mann‒Whitney *U* test was used for comparisons, and *p* < 0.05 indicated statistical significance. A delay of 0.95 points in dental development and delayed exfoliation of primary dentition between 1.31 and 1.66 years were described in the study group. A root resorption delay of 11.8% was described among the 5 primary teeth of 23.3% among the single-rooted teeth and of 5.6% among the two-rooted teeth in children with OI medicated with BFs (*p* < 0.05). Delayed alveolar eruption of the first permanent molar at 0.31 years of age was found in children with OI medicated with BFs. We detected delayed tooth development at one stage of maturation in the study group, which was clinically imperceptible. The dental age (≤ 0.55 years) was greater than the chronological age in both groups. We also reported delayed exfoliation of the primary dentition (from 1.31 to 1.66 years), delayed root resorption of the primary dentition (11.8%), and delayed (from 1 mm to 1.25 mm) root resorption of the primary molars in the study group. Although the degree of dental development of the first permanent molar was similar between the two groups, we found delayed (0.31 years) alveolar eruption in the study group and a greater delay (0.44 years) in children whose cumulative dose of bisphosphonates exceeded 2000.

## Introduction

Osteogenesis imperfecta (OI), or brittle bone disease, is a group of heterogeneous disorders characterized by bone fragility, multiple fractures, skeletal deformity, short stature, low bone mass, and other connective tissue manifestations [1]. In most patients, the disorder is caused by mutations in one of the two genes encoding collagen type I, but in some individuals, no such mutations are detectable [2]. The diagnosis of OI usually depends on family history and clinical presentation characterized by a fracture (or fracture) during the prenatal period, at birth or in early childhood; genetic tests can confirm the diagnosis [3].

OI is a rare disease, with an incidence ranging between 6 and 20 cases per 100000 live births [2]. There are patients who experience mild symptoms of the disease without being diagnosed [1].

Among the orofacial alterations in patients with OI, dentinogenesis imperfecta (DI) type I has received considerable research attention, whereas eruptive alterations have only recently been the subject of several publications. Meanwhile, eruptive chronological delays have received some attention, such as the studies of Kamoun-Goldrar et al. [4] and the study of Del Rio-Cantero et al. [5]. This also includes the study of the retention of primary dentition conducted by Vuorimies et al. [6].

Bisphosphonates (BPs) are indicated for children with OI. Their main mechanism of action is to inhibit osteoclast function and bone resorption [7]. The eruptive process is carried out at the expense of alveolar bone resorption for teeth that do not have primary teeth and for those that do have it at the expense of root resorption of the primary teeth. Osteoclasts are responsible for the resorption of both processes [8]. Studies in experimental animals have shown that BP alters the eruptive process [9, 10].

Any alteration that causes any deviation in dental development will be of interest to pediatric dentists, orthodontists, pediatricians, endocrinologists, and other professionals who care for this group. When such deviations are potentially related to our dental treatments, they can contribute to more unique adaptations of these treatments, when possible. Therefore, the aim of this investigation was to study the eruptive process (dental development of permanent dentition, resorption of the primary dentition, and alveolar eruption of the first permanent molar) in children with OI medicated with BPs and to compare the results with those of a control group.

## Materials and Methods

The study was carried out in the Department of Clinical Dental Specialties of the Faculty of Dentistry of the Complutense University of Madrid (UCM). The dental clinic of the Master of Pediatric Dentistry of the UCM treats patients on a voluntary basis for comprehensive dental treatment, thus having a homogeneous group of patients with OI. The research was carried out in strict compliance with the ethical and legal standards that govern biomedical research in Spain. Specifically, it has been duly accredited by the Research Ethics Committee of the corresponding center (San Carlos Clinical Hospital in Madrid).

### Study Group

The study group consisted of 34 panoramic radiographs pertaining to children between 6 and 11.9 years of age with a confirmed diagnosis of OI upon treatment with BPs. Since OI is a rare disease, an adequate sample size calculation could not be reached; therefore, studies such as those by Vuorimies et al. [6] and Del Rio-Cantero et al. [5] were used as references. The inclusion criteria of the study group are described below:

- OI diagnosis.
- Children aged 6 through 11.9 years.
- Detailed medical report concerning the type of OI, type of antiresorptive medication, child weight, and administration period.
- Antiresorptive administration period equal to or greater than 1 year.
- Informed consent of the guardian or legal representative.
- In the case of agenesis of 3.1, 3.2, 3.3, 3.4, 3.5, 3.6, and 3.7, contralateral teeth 4.1, 4.2, 4.3, 4.4, 4.5, 4.6, and 4.7 must be present.
- Availability of panoramic radiograph.

The exclusion criteria of the study group are described below:

- Absence of data regarding the date of X-ray collection.
- Distorted and/or uncalibrated X-rays.
- Pulp treatments at 7.1, 7.2, 7.3, 7.4, and 7.5.
- Patients undergoing orthodontic treatment.
- Bilateral agenesis in quadrants III and IV.

### Control Group

The control group sample was similar to the study group in terms of age and sex to enable comparisons. A total of 367 panoramic radiographs of children between 6 and 11.9 years of age without systemic diseases who received comprehensive dental treatment at the UCM were obtained.

### Radiographic Analysis

Two examiners evaluated the clinical histories and panoramic radiographs of patients from the Department of Clinical Dental Specialties at the UCM. For the study of the main research topics (dental development-resorption), widely proven methodologies have been selected and used in other specific studies on both topics.

The dental development of 3.1, 3.2, 3.3, 3.4, 3.5, 3.6, and 3.7 was established via the method of Demirjian et al. This method is based on the progression of mineralization of each tooth, and the shape does not depend on the length estimates [11]. Eight stages are recognized for molars and premolars (from stage A to stage H), and 6 stages are recognized for incisors and canines (from stage C to stage H). Stage A corresponds to the mineralization of the cusps in the form of one or more cones without fusion, whereas stage H corresponds to a fully developed tooth [11].

First, the stages of each tooth were calculated, which were later converted into dental maturation scores. Once the degree of dental maturation of the 7 teeth was calculated, the sum of the degree of dental maturation of the 7 teeth was calculated. In summary, the dental age of each patient was calculated via the estimates of Demirjian et al. [11] and Feijóo et al. [12].

The alveolar eruption of the first lower molar was studied radiographically. The alveolar eruption of the molar was considered when it pierced the cortical bone, regardless of whether it had torn the oral mucosa, as studied by other authors [6]. As reference values, we took the published norms for the time of tooth eruption of healthy Finnish children, since there is no information available on this subject in our population. The time of tooth eruption was considered altered if it was outside the 5th or 95th percentile [13].

Root resorption of the primary dentition of the third quadrant was studied via two methods: the method of Haavikko et al. [14] and the use of software that measures the pixels indicated in the image after X-ray calibration. Each tooth was assigned the following classification: 1: No root resorption; 2: ¼ part of the root is resorbed; 3: Half of the root is resorbed; and 4: ¾ of the root is resorbed or exfoliated. For single-rooted teeth, such as 7.1, 7.2, and 7.3, a value was assigned to each tooth according to the stage. For birooted teeth, such as those with scores of 7.4 and 7.5, a stage was assigned for each root (mesial and distal), resulting in a maximum score value of 28 points. Since the roots of biradicular teeth are not resorbed symmetrically, the roots were studied separately [15]. To objectively measure the root resorption of primary molars, we used software that measures the pixels indicated in the image. To obtain the measurement in millimeters, it was necessary to obtain the relationship between the pixel size and mm.

Patients had received bisphosphonate (BP) for a period equal to or greater than 1 year. Pamidronate and zoledronate are the two types of BPs that the study group has received. These BPs have different antiresorptive effects. The relative potencies of different BPs were established as follows: 100 for pamidronate and 10000 for zoledronate [16]. The cumulative dose of BP was obtained by calculating the total dose received (mg)/weight (kg) and multiplying by the relative potency of the medication.

The distributions of the groups according to sex and age are shown in Table 1. Among the 488 children initially included in the study, 87 were excluded after applying the exclusion criteria; thus, 401 children were included. The 401 children were distributed as follows: 34 children in the study group and 367 children in the control group. The study group (*n* = 34) included 18 girls with a mean age of 8.43 years and 16 boys with a mean age of 8.44 years. In the control group (*n* = 367), there were 169 females with a mean age of 9.39 years and 198 males with a mean age of 9.02 years.

**Table 1:** Distribution of the study group and the control group according to sex and age

Age groups	Study group *n* (%)		Control group *n* (%)		Total *n* (%)
	Females	Males	Females	Males	
10–10.99	3 (16.7%)	–	31 (18.3%)	31 (15.7%)	65 (16.2%)
11–11.99	2 (11.1%)	3 (18.8%)	30 (17.8%)	35 (17.7%)	70 (17.5%)
6–6.99	5 (27.8%)	4 (25.0%)	5 (3.0%)	37 (18.7%)	51 (12.7%)
7–7.99	3 (16.7%)	4 (25.0%)	35 (20.7%)	30 (15.2%)	72 (18.0%)
8–8.99	3 (16.7%)	3 (18.8%)	32 (18.9%)	33 (16.7%)	71 (17.7%)
9–9.99	2 (11.1%)	2 (12.5%)	36 (21.3%)	32 (16.2%)	72 (18.0%)
Total	18 (4.5%)	16 (4.0%)	169 (42.1%)	198 (49.4%)	401 (100%)

The majority of patients in the study group (61.8%) received a dose of BPs ≥ 2000, while the remaining 38.20% received a cumulative dose of BPs < 2000.

### Statistical Analysis

The statistical analysis was carried out via the computer application IBM-SPSS Statistics version 25 (reference: IBM Corp. Released 2017. IBM-SPSS Statistics v 25.0 for Windows; Armonk. NY. USA). Student’s *t* test, the Mann‒Whitney *U* test, the Wilcoxon test, and the Kruskal‒Wallis test were used. In all cases, the effect size value (on the R^2^ scale) was calculated as a way of assessing the magnitude of the differences. In all these inferential statistical tests, *p* < 0.05 was considered significant, and *p* < 0.01 was considered highly significant.

Some figures were obtained via Python programming. The tabulation, sorting, and cleaning of the database were performed via Microsoft Excel [reference: Microsoft Excel. (2023). Windows. Microsoft]. The ROC curve methodology was used to determine appropriate cut-off points on the basis of the sensitivity values, positive predictive value and the Youden index.

From the total sample of the study group (*n* = 34), 20 cases (58.8%) were randomly selected to obtain a minimum N with sufficient statistical strength. A proportional criterion of a 2:1 ratio of subjects from the control group to subjects from the study group was chosen; thus, 40 subjects were again randomly selected from among the 367 participants in the control group (10.9%).

## Results

Sixty subjects (20 from the study group and 40 from the control group) were selected via the random sampling function implemented in SPSS statistics within their menus. This subsample (*N* = 60) was studied at two different times by the same evaluator (intraobserver reliability) and by 2 different evaluators (interobserver reliability). No differences in any of the average variables reached statistical significance (*p* > 0.05); that is, there were no differences between the measurements made by observer B with respect to those of observer A. In addition, the correlation coefficients (which can be considered reliability coefficients) that were obtained were so high that they bordered perfection (*r* = 1; maximum reliability). Therefore, there was very solid statistical evidence of a high degree of interobserver reliability and that the values recorded were independent of who made the measurements.

### Dental Development, Dental Age, and Chronological Age

#### Children with OI and Healthy Children

The average age of the 34 children with OI was 8.43 years (± 1.77) and that of the 367 children in the control group was 9.19 years (± 1.62). The children in the control group were 0.76 years older than the children in the study group, as described in Table 2.

**Table 2:** Age of the study group and the control group

		(*n*)	Average (age)	Standard deviation	Median	25th percentile	75th percentile
Age	Control group	367	9.19	± 1.62	9.33	7.83	10.39
	Study group	34	8.43	± 1.77	8.10	6.88	9.70
	Total	401	9.13	± 1.65	9.24	7.78	10.35

The dental development of the 34 children in the study group reached a mean score of 76.63 (± 16.52), whereas the dental development of the 367 children in the control group reached a mean score of 84.48 (± 12.84); thus, there was a difference of 7.85 points between the groups. When the age difference between the groups was equal, a difference in dental development of 0.95 points was described, which indicates a delay of 0.95 points in dental development in children with OI medicated with BPs. The difference was small but statistically significant (*p* = 0.003) (Table 3).

**Table 3:** Dental development of the study group and the control group

		(*n*)	Average (age)	Standard deviation	Median	25th percentile	75th percentile
Dental Development	Control group	367	84.48	± 12.84	88.20	81.20	93.70
	Study group	34	76.63	± 16.52	80.00	59.90	90.40
	Total	401	83.81	± 13.35	88.20	80.20	93.50

A global analysis of the estimated dental age based on the tables of Demirjian et al. and chronological age revealed an advance in dental age of 0.34 years in the study group and 0.55 years in the control group.

The estimated dental age based on the tables of Feijóo et al. and the chronological age were also analyzed in the same way. In this case, the dental age was close to the chronological age, with only a slight delay of 0.008 years in the study group and 0.17 years in the control group.

Table 4 summarizes the differences between the two groups. A very slight delay in dental development was described in children with OI, which is imperceptible clinically, and although this difference was small, it was statistically significant. With respect to the differences between chronological age and dental age, these differences were less than ± 0.55 years in all the groups analyzed; therefore, dental age was very close to chronological age and both the estimates of Demirjian et al. and Feijóo et al. were close to chronological age.

**Table 4:** Comparative summary of chronological age and dental age between groups

		Chronological age	Dental age	Difference	Delay or advancement of dental age
Age estimation according to Demirjian et al.	Control group	9.19	9.74	0.55	Advancement of 0.55 years of dental age in the control group
	Study group	8.43	8.77	0.34	Advancement of 0.34 years of dental age in the study group
Age estimation according to Feijóo et al.	Control group	9.19	9.36	0.17	Advancement of 0.17 years in dental age in the control group
	Study group	8.43	8.35	−0.008	Delay of 0.008 years in dental age in the study group

The average age of the 26 children with OI type I was 8.54 years (± 1.75) and that of the 8 children with OI types III–IV was 8.11 years (± 1.92). The 26 children with OI type I reached a dental development of 77.59 (± 15.02), and the 8 children with OI types III–IV reached a dental development of 73.5 (± 21.58), indicating a delay of 4.09 points in dental development in children with OI types III–IV compared with the OI type I group, but the difference did not reach statistical significance.

An advanced dental age of 0.23 years was described in the OI type I group and 0.67 years in the OI types III–IV group, according to the estimates of Demirjian et al. Based on the estimates of Feijóo et al., a delay of 0.13 years was described in the OI type I group and 0.07 years in the OI types III–IV group.

Children with OI were analyzed in two subgroups on the basis of the cumulative dose of bisphosphonates (CDBPs). One group comprised 13 (38.2%) children who received < 2000 cumulative doses of BPs, and the other group comprised 21 (61.8%) children who received ≥ 2000 cumulative doses of BPs.

There were no differences in dental development, dental age, or chronological age based on the cumulative dose of BPs in each child with OI. In terms of the differences between chronological age and dental age, according to Demirjian et al., there was an increase in the dental age of 0.30 years in children with < 2000 CDBPs and 0.36 years in children with ≥ 2000 CDBPs. However, with the estimation of Feijóo et al., a delay of 0.01 years was described in children with < 2000 CDBPs and a delay of 0.13 years in children with ≥ 2000 CDBPs.

### Alveolar Eruption of the First Permanent Molar

A high percentage of alveolar eruption of the first permanent molar was described in all types of OI, with 96.2% of the patients having type I OI and 87.5% of the patients having types III–IV OI. In only two cases alveolar eruption did not occur in 3.6. Among the different types of OIs, eruption of the first molar occurs when it is in stage G.

Alveolar eruption in children with OI with < 2000 CDBPs occurred at 6.23 years, and it occurred at 6.67 years in children with OI ≥ 2000 CDBPs. A delay of 0.44 years in alveolar eruption of 3.6 was described in children who had received more CDBPs. Statistical significance could not be calculated since the results are an estimation of the ROC curve analysis.

#### Children with OI and Healthy Children

An alveolar eruption of the first permanent molar in children with OI occurred at 6.63 years, and it occurred at 6.32 years in children in the control group, thus indicating a delay of 0.31 years in children with OI. Statistical significance could not be calculated since the results are an estimate of the ROC curve with a Youden index of almost 1.

An analysis of the dental development of the first permanent molar in both groups revealed that alveolar eruption occurred when it reached the G development stage; therefore, no differences were described between the groups.

### Root Resorption of Primary Dentition

The exfoliation of the 5 primary teeth in children with OI treated with BPs occurred at 11.16 years of age, and it occurred at 9.5–9.85 years of age in healthy children, with a delay in the exfoliation of the primary dentition of 1.31–1.66 years. The age at which the 3 single-rooted teeth exfoliate in children with OI was 10.58 years. However, in the control group, exfoliation of single-rooted teeth occurred between 8.88 and 9.24 years, as shown in Table 5. These values were obtained by calculating the ROC curve using different cut-off points for chronological age, with a Youden index close to 1.

**Table 5:** Age at which exfoliation of single- and double-rooted teeth occurs in children with OI and in healthy children

Exfoliation	Study Group	Control Group	Difference
Complete Temporary Dentition	11.16 (years)	9.50–9.85 (years)	1.31–1.66 (years)
Single Root Teeth (7.1, 7.2 Y 7.3)	10.58 (years)	8.82–9.24 (years)	1.34–1.76 (years)
Biradicular Teeth (7.4 Y 7.5)	11.16 (years)	9.5–9.7 (years)	1.46–1.66 (years)

In terms of root resorption of primary dentition via the qualitative method, less root resorption was described in children with OI, with 5 points less in the root resorption of the 5 temporary teeth (*p* = 0.001), 1.47 points less in the root resorption of the 3 single-rooted teeth (*p* = 0.001), and 3.56 points less in the root resorption of the 2 double-rooted teeth (*p* = 0.001). Once the ages were equalized, a delay of 3.32 points (11.8%) was described in the root resorption of the 5 temporary teeth, 0.9 points (5.6%) in the root resorption of the single-rooted teeth, and 2.8 points (23.3%) in the root resorption of the double-rooted teeth in children with OI; these differences were statistically significant, as described in Table 6.

**Table 6:** Comparative synthesis of root resorption in the study group and the control group via the qualitative method

	Control Group	Study Group	Difference	Difference once the ages are equal	(*p*)	Delay or advance
Root resorption of the 5 primary teeth	21.53 points	16.53 points	5 points	3.32 points	0.001	Delay of 3.32 points (11.8%) in the RR of the 5 temporary teeth in children with OI medicated with BPs
Root resorption of the 3 single-rooted teeth	10.56 points	9.09 puntos points	1.47 points	0.9 puntos	0.001	Delay of 0.9 points (5.6%) in the RR of the 3 single-rooted teeth in children with OI treated with BPs
Root resorption of the 2 birooted teeth	11 points	7.44 points	3.56 points	2.8 points	0.001	Delay of 2.8 points (23.3%) in the RR of the 2 birooted teeth in children with OI treated with BPs

In terms of root resorption of temporary dentition via the quantitative method, a delay of 1.31 mm in root resorption of the mesial root of 7.4 (*p* = 0.001) and a delay of 1.34 mm in the distal root of 7.4 in children with OI (*p* = 0.001) was described. A delay of 1.63 mm in the root resorption of the mesial root (*p* = 0.001) and a delay of 1.25 mm in the distal root of 7.5 (*p* = 0.041) was also described in children with OI, and these differences were statistically significant.

The mean mesial and distal root lengths of 7.4 and 7.5 tended to decrease in healthy children as age increased. However, in children with OI, there were notable variations in root length. This discrepancy could indicate an alteration in the root resorption process in children with OI.

## Root Length of the First Mandibular Primary Molar (7.4)

A delay of 1.31 mm in the root resorption of the mesial root of 7.4 and a delay of 1.34 mm in the root resorption of the distal root of 7.4 in children were described. Once the ages were equalized in both groups, a delay in the root resorption of the mesial root of 1.01 mm (*p* < 0.001) and a delay in the distal root of 1.02 mm (*p* < 0.001) of 7.4 in children with OI medicated with BPs were found, with statistical significance.

## Root Length of the Lower Second Primary Molar (7.5)

An analysis of the differences revealed delays of 1.63 mm in the mesial root and 1.25 mm in the distal root. Once the ages in both groups were equalized, a delay in root resorption of 1.25 mm (*p* < 0.001) in the mesial root and 0.79 mm (*p* = 0.041) in the distal root was described for 7.5 in children with OI medicated with BPs.

A comparative summary of the root resorption of the mesial and distal roots at 7.4 and 7.5 between the groups via the quantitative method is presented in Table 7.

**Table 7:** Comparative synthesis of root resorption of the mesial and distal roots at 7.4 and 7.5 between groups via the quantitative method

	Control Group	Study Group	Difference	Difference once the ages are equal	(*p*)	Delay or advance
MRL 7.4	2.05 mm	3.36 mm	1.31 mm	1.01 mm	< 0.05	Delay in RR of 1.01 mm in the mesial root of 7.4 in children with OI medicated with bisphosphonates
DRL 7.4	2.12 mm	3.46 mm	1.34 mm	1.02 mm	< 0.05	Delay in RR of 1.02 mm in the distal root of 7.4 in children with OI treated with bisphosphonates
MRL 7.5	3.23 mm	4.86 mm	1.63 mm	1.02 mm	< 0.05	Delay in RR of 1.25 mm in the mesial root of 7.5 in children with OI medicated with bisphosphonates
DRL 7.5	3.95 mm	5.20 mm	1.25 mm	0.79 mm	< 0.05	Delay in RR of 0.79 mm in the distal root of 7.5 in children with OI treated with bisphosphonates

*MRL* Mesial radicular length, *DRL* Distal radicular length

## Discussion

Taqi y cols et al. [17] described a higher prevalence of agenesis in patients with OI than in the general population. This clinical finding was associated with the early administration of BPs, which seems to increase the risk of developing dental alterations. In this study, children with bilateral agenesis in the lower arch were excluded, and the prevalence of alterations in the number and size of teeth in the groups studied therefore could not be calculated. Dental agenesis and morphological aberrations of the dentition described in patients with OI, as well as enamel defects, are associated with the early initiation of the administration of BPs, specifically before 2 years of age [18].

We found a delay of 0.95 points in dental development. Notably, this difference is within the 25th and 75th percentiles of the healthy children analyzed in this study. An analysis of the type of OI and the cumulative dose of BPs revealed a delay in dental development in children with OI types III–IV and in children who received more medication, but the difference was not statistically significant. Malmgren et al. reported differences between the different types of OI, indicating that the dental development of children with OI types III and IV was delayed compared with that of their control groups [19].

Chronological age and dental age were calculated on the basis of the estimation of Demirjian et al. [11], who reported an advance in dental age of 0.34 years in the study group and 0.55 years in the control group. This is consistent with the results of Moca et al., who indicated that the Demirjian method overestimates dental age [20]. However, if we analyzed by age subgroup, a dispersed pattern was observed in the study group, but an advance in dental age was generally described. Many authors have indicated that the method of Demirjian et al. tends to overestimate dental age, and for this reason, they have adapted the tables of Demirjian et al. to the populations studied. Importantly, the method of Demirjian et al. [11] was calculated on the basis of the French–Canadian population, which is different from the Spanish population. It is also not the only method for determining dental age; there are other methods, such as those described by other authors [21], but the method of Demirjian et al. is the most commonly used method in the scientific literature [11]. The authors described an overestimation of dental age with this method of between 0.46 and 1.73 years in females and between 0.15 and 2.02 years in males [20].

Feijóo et al. adapted the estimation of Demirjian et al. to the Spanish population; for this reason, we also calculated the dental age with this method, which is the first study that has calculated the dental age of children with OI with this method. When we analyzed the dental age according to the estimates of this author, we found that the delay of dental age was 0.008 years in children with OI. These results agree with the results obtained by authors, such as Malmgren et al. [19], who reported that antiresorptive medication delays the dental age of children with OI. Other authors, such as Vuorimies et al. [6], considered a delay or an advancement of the dental age only if this difference exceeded ± 12 months. If we consider this criterion in this study, no differences would be described between dental age and chronological age, since the differences were within the range ± 12 months. Malmgren et al. [19] did not consider this criterion or at least did not describe it in their methodology. Perhaps, this is why both authors differed in their results, although Malmgren et al. [19] reported that the difference between their results and those of Vuorimies et al. [6] lies in the fact that most of the children with OI in their study received antiresorptive medication before 2 years of age, as in this study.

The eruption of permanent teeth requires root resorption of temporary teeth and, consequently, their exfoliation. BPs act by blocking the action of osteoclasts and, therefore, decreasing bone resorption [22]. In vitro studies have shown a direct association between BPs and a reduction in bone resorption by osteoclasts [10]. In the present study, we found a delay of 0.31 years in the alveolar eruption of the first permanent molar in children with OI treated with BPs and a delay of 0.44 years in children who received the most medication (≥ 2000 CDBPs).

We found a delay in root resorption and alveolar eruption of the first permanent molar in children with OI types III–IV compared with children with OI type I, but the difference did not reach statistical significance. These results could be biased by the small sample size. Another possible explanation could be that there were cases in which patients with OI types III–IV were considered OI type I due to the positive effect of medication; thus, analyzing OI types is complicated and can create confusion. Owing to medication, severe forms of OI have become milder.

Authors who have studied the mesial and distal roots separately reported that, in 36% of cases, asymmetric root resorption of primary molars occurs [23]. To avoid confusion in this study, we studied the mesial and distal roots of primary molars separately via the method established by Haavikko et al. [14].

We found a delay in the exfoliation of the primary dentition of between 1.31 and 1.66 years in children with OI treated with BPs, an estimate obtained via ROC curve analysis. These results support the results of Kamoun-Goldrat et al. [4], who described a delay of 1.67 years in eruption. Other authors, such as Malmgren et al. [19] reported a delay in dental eruption in children with OI, and Del Rio Cantero et al. [5] reported a delay in the eruption of the first phase mixed dentition in children with OI, which was related directly to the accumulated dose of BPs. A group of children with OI without antiresorptive treatment is not available. However, root resorption has been analyzed on the basis of the cumulative dose of BPs and a greater delay in root resorption has been found in children with a higher accumulated dose of bisphosphonates, although the difference has not reached statistical significance.

When we studied root resorption in males and females within the same group, we found that males in the control group were delayed compared with females. However, in the study group, we found no differences between females and males. This could be because the disease or medication could have a homogenizing effect on root resorption in females and males with OI.

When we analyzed root resorption by subgroups based on sex, we detected a delay in both females and males with OI. These results agree with the results of Malmgren et al. [19] and with those of a recent study published by our research team [24]. However, with respect to the root resorption of birooted teeth, specifically 7.5, we found no difference in males. Alterations in dental development, such as DI, dentine dysplasia, or endocrine alterations, could affect root resorption in primary teeth [25].

From a professional point of view, it is necessary and our responsibility to identify early pathological consequences of OI in the oral and orofacial spheres and those derived from changing treatment protocols to establish an intervention plan adapted to age and with implications for affected people to limit their pathology.

## Conclusion

We detected delayed tooth development at one stage of maturation in the study group, which was clinically imperceptible. The dental age (≤ 0.55 years) was greater than the chronological age in both groups. We also reported delayed exfoliation of the primary dentition (from 1.31 to 1.66 years), delayed root resorption of the primary dentition (11.8%), and delayed (from 1 to 1.25 mm) root resorption of the primary molars in the study group. Although the degree of dental development of the first permanent molar was similar between the two groups, we found delayed (0.31 years) alveolar eruption in the study group and a greater delay (0.44 years) in children whose cumulative dose of bisphosphonates exceeded 2000.

## Abbreviations

OI: Osteogenesis Imperfecta
BPs: Bisphosphonates
RR: Root Resorption
DD: Dental Development
CDBPs: Cumulate dose of biphosphonates
DI: Dentinogenesis Imperfecta
